# Role of Telomerase Reverse Transcriptase in Glial Scar Formation After Spinal Cord Injury in Rats

**DOI:** 10.1007/s11064-013-1097-x

**Published:** 2013-06-22

**Authors:** Xu Tao, Yang Ming-kun, Sheng Wei-bin, Guo Hai-long, Kan Rui, Tu Lai-yong

**Affiliations:** Department of Spinal Surgery, First Affiliated Hospital of Xinjiang Medical University, Urumqi, China

**Keywords:** Spinal cord injury, Glial scar, Telomerase reverse transcriptase

## Abstract

The study aims to determine the expression of telomerase reverse transcriptase (TERT) in the glial scar following spinal cord injury in the rat, and to explore its relationship with glial scar formation. A total of 120 Sprague–Dawley rats were randomly divided into three groups: SCI only group (without TERT interference), TERT siRNA group (with TERT interference), and sham group. The TERT siRNA and SCI only groups received spinal cord injury induced by the modified Allen’s weight drop method. In the sham group, the vertebral plate was opened to expose the spinal cord, but no injury was modeled. Five rats from each group were sacrificed under anesthesia at days 1, 3, 5, 7, 14, 28, 42, and 56 after spinal cord injury. Specimens were removed for observation of glial scar formation using hematoxylin-eosin staining and immunofluorescence detection. mRNA and protein expressions of TERT and glial fibrillary acidic protein (GFAP) were detected by reverse-transcription (RT)-PCR and western blotting, respectively. Hematoxylin-eosin staining showed evidence of gliosis and glial scarring in the spinal cord injury zone of the TERT siRNA and SCI only groups, but not in the sham group. Immunofluorescence detection showed a significant increase in GFAP expression at all time points after spinal cord injury in the SCI only group (81 %) compared with the TERT siRNA group (67 %) and sham group (2 %). In contrast, the expression of neurofilament protein 200 (NF-200) was gradually reduced and remained at a stable level until 28 days in the SCI only group. There were no NF-200-labeled cells in the spinal cord glial scar and cavity at day 56 after spinal cord injury. NF-200 expression at each time point was significantly lower in the SCI only group than the TERT siRNA group, while there was no change in the sham group. Western blotting showed that TERT and GFAP protein expressions changed dynamically and showed a linear relationship in the SCI only group (r = 0.765, *P* < 0.01), while there was no obvious linear relationship in the sham group (r = 0.208, *P* = 0.121). RT-PCR results showed a dynamic expression of TERT and GFAP mRNA in the SCI only group, exhibiting a linear relationship (r = 0.722, *P* < 0.01), while there was no linear relationship in the sham group (r = 0.206, *P* = 0.180). Our data indicate that TERT has a dynamic expression in the spinal cord glial scar, which positively correlates to GFAP expression, and may be important for promoting glial scar formation.

## Introduction

Spinal cord injury can induce a series of severe motor, sensory, and autonomic dysfunctions in humans. Formation and development of glial scarring is the predominant pathological change following spinal cord injury [1]. Experimental studies have shown that various interventions for spinal cord injury are unable to penetrate the glial scar barrier to prevent glial scar formation. Astrocytes play a critical role in glial scar formation and inhibition of axonal regeneration [2, 3].

After spinal cord injury, astrocytes become activated and show reactive hyperplasia, hypertrophy and proliferation, and increased glial fibrillary acidic protein (GFAP) expression and cell division [4, 5].

In addition to protecting the telomere, telomerase reverse transcriptase (TERT) has multiple non-telomere length-dependent effects that are closely associated with cell activation, proliferation, and apoptosis [6–9]. Some researchers reported that excitatory and traumatic brain injury could induce telomerase expression in microglia by activating a series of cytokines and growth factors, thereby promoting glial scar formation [10]. However, the role of TERT in the activation and proliferation of astrocytes after spinal cord injury, and glial scar formation and development remains unclear. Thus, in the present study we examined the effects of TERT on glial scar formation after spinal cord injury in rats.

## Materials and Methods

### Main Reagents and Instruments

The reverse transcription (RT)-PCR TERT assay kit was purchased from Roche (St. Louis, MO, USA). The GFAP detection kit, FITC-conjugated goat anti-rabbit antibody, mouse anti-GFAP antibody, rabbit anti-GFAP antibody, and neurofilament protein 200 (NF-200) were purchased from Sigma. The plasmid vector PBCA-NPS-TERT was provided by Invitrogen (Carlsbad, CA, USA). The PCR instrument was from BioRad Inc. (Berkeley, CA USA).

### Spinal Cord Injury Model

A total of 120 male Sprague–Dawley rats (clean grade, 180–220 g) were purchased from the Experimental Animal Center, Xinjiang Medical University (license No. SYXK (Xin) 2003-001). The rats were randomized into SCI only group, TERT siRNA group, and sham group (n = 40 rats per group). All rats were anesthetized with a combination of 2 mL ketamine, 1 mL atropine, 2 mL diazepam, and 5 mL 0.85 % sodium chloride via intraperitoneal injection (0.5 mL/100 g). After anesthesia, the modified Allen’s weight drop method was used to induce spinal cord injury at the T8 segment in the TERT siRNA and SCI only groups under aseptic conditions [11]. After modeling, the bleeding was stopped and the incision sutured, and the bladder was manually squeezed every 8 h to help urination until spontaneous voiding. In the sham group, the spinal cord was exposed but not damaged. On the basis of GenBank bioinformatics analysis, we designed sense and antisense nucleotides targeting astrocyte TERT mRNA in rats (antisense oligodeoxynucleotide 5′-GTTAGGGTTAG-3, sense oligodeoxynucleotide 5′-CTAACCCTAAC-3′. In this study, we used PBCA-NPs-TERT to effectively inhibit TERT expression. In the TERT siRNA group, PBCA-NPs-TERT saline solution (91.861692 pmol/μL) was injected at a dose of 50 μg/kg body mass into the surgical site at 30 min after modeling, twice per day. No administration was performed in the SCI only groups and sham groups.

### Sample Collection

In each group, five rats were sacrificed under anesthesia at days 1, 3, 5, 7, 14, 28, 42, and 56. Under sterile conditions, 50 mg of spinal cord tissue, 5 mm in length, was taken from the central zone of the injured spinal cord, and was then rinsed with 0.1 % DEPC water and stored in nuclease-free cryovials at −80 °C.

### Pathological Observation of Spinal Cord Glial Scar

#### Hematoxylin-Eosin Staining

Two sections from rats were taken at each time point for hematoxylin-eosin staining to observe the morphology of spinal cord tissues, changes in glia and nerve cells, and glial scar hyperplasia.

#### Immunofluorescence Detection of GFAP and NF-200 Expression

Frozen sections were rinsed with 3× PBS (once for 10 min), blocked with 10 % goat serum for 1 h at room temperature, and incubated at 4 °C overnight. Sections were rinsed with 3× PBS (once for 10 min), and incubated in the following primary antibodies: chicken anti-GFAP/NF-200 polyclonal antibody (1:2,000), mouse anti-CDllb/c monoclonal antibody (1:200), and rabbit anti-Fibronectin polyclonal antibody (1:200), for 24 h at 4 °C. Sections were then rinsed with 3× PBS (pH 7.4; once for 5 min), followed by incubation with secondary antibodies (DyLight488 green fluorescence-labeled goat anti-mouse IgG; Texas Red-labeled rabbit anti-chicken IgY) for 1 h in the dark at 37 °C. After 3× PBS rinses (once for 5 min), the sections were observed under a 200× fluorescence microscope. Numbers of GFAP and NF-200-positive cells in five fields of view were counted and the area of positive cells was calculated using Image-ProPlus 6.0 software (Media Cybernetics Inc., Rockville, MD, USA).

### Western Blotting Assay for Detection of TERT and GFAP Expression

Total protein extract from approximately 50 mg of spinal cord scar tissue was prepared from the various time points. Protein samples (20 μL) from each group were resolved in a 7.5 % sodium dodecyl sulfate polyacrylamide gel electrophoresis (SDS-PAGE) gel and transferred onto film. The samples were incubated in rabbit anti-rat TERT monoclonal antibody (1:100) for 2 h at 37 °C, and then stored at 4 °C overnight. After incubation with horseradish peroxidase-labeled goat anti-rabbit IgG (1:50) for 1 h at room temperature, the samples were colored with chromogenic substrate for 2 min in the dark. Glyceraldehyde-3-phosphate dehydrogenase (GAPDH) served as the internal control, and a gel imaging analysis system was employed for semi-quantitative analysis. The ratio of telomerase to GAPDH was calculated.

### Detection of TERT and GFAP mRNA by RT-PCR

Total RNA was extracted using the Trizol method according to the manufacturer’s instructions (Invitrogen). Primers sequences used were: TERT: upstream, 5′-TCCGCACGTT GGTTGCCCAG-3′, downstream, 5′-CCTCTCACCGCGCTCGCA AA-3′ (product size = 203 bp); GADPH: upstream, 5′-GCTCTCTGCTCCTCCCTGTTCT-3′, downstream, 5′-CAGGCGTCCG ATACGGCCAAA-3′ (product size = 450 bp); GFAP: upstream, 5′-CTG AATTC TGCTGGCTTCAAGG-3′, downstream, 5′-CTAAGCTTGCTCTGCGTTGCGG-3′ (product size = 624 bp); and β-actin: upstream, 5′-GCGGGAAATCGTGCTGA CATT-3′, downstream, 5′-GATGGAGTTGA AGGTAGTTTCGTG-3′ (product size = 314 bp). PCR was performed as follows: denaturing at 94 °C for 30 s, annealing at 55 °C for 30 s, extension at 72 °C for 30 s, for 35 cycles, followed by extension at 72 °C for 5 min. PCR products were analyzed on a 15 g/L agarose gel. TERT and GFAP mRNA expressions were confirmed by comparing the PCR band intensity between TERT and GAPDH as well as between GFAP and β-actin. All gene expression data were calculated as 2^−△△Ct^.

### Statistical Analysis

Data are expressed as mean ± SD, and were analyzed using SPSS 19.0 (IBM, Armonk, NY, USA). Analysis of variance for a completely randomized design was used to compare differences between the three groups. A least significant difference test was used for comparison between groups. *P* < 0.05 was considered statistically significant.

## Results

### Quantitative Analysis of Experimental Animals

Four rats died during the modeling and six died after modeling, which were supplemented with new rats. No infection occurred after modeling. A total of 120 rats were included in the final analysis.

### Transfection Efficiency of the Plasmid Vector

Negative and false-positive tests showed that the inhibition rate of the constructed plasmid vector was 81.05 ± 7.65 %.

### Results for the Hematoxylin–Eosin Staining

**Fig. 1:**
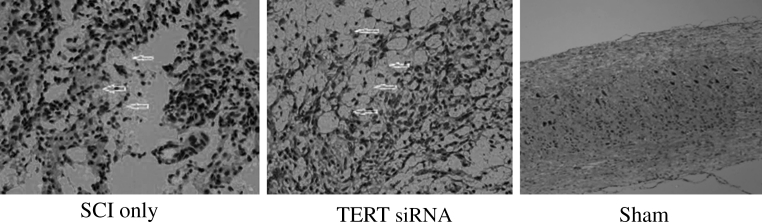
Astrocytes proliferation and glial scars formation (*arrows*) at 28 days after spinal cord injury in the telomerase reverse transcriptase (TERT siRNA) group and SCI only group. The responses of the TERT siRNA group were greater than in the SCI only group. No astrocyte proliferation or glial scar formation were seen in the sham group (×200)

In the SCI only group, there were few hemorrhagic foci in the spinal cord gray matter and white matter, severe damage to the structure of the spinal cord, dissolved gray matter neurons, a large necrotic area, and cyst formation. A great amount of axons and vacuoles could be seen in the white matter, and nerve fibers were disordered. By the end of day 28, the hemorrhagic foci had practically disappeared in the spinal cord gray matter and white matter, and the structure of the spinal cord was damaged to a severe extent. There was further death of gray matter neurons, which formed a large number of vacuoles. In addition, inflammatory infiltration was alleviated, and a large number of glial cells surrounded the cavity to form a dense glial scar. In the TERT siRNA group, the spinal cord also appeared to have visible structural damage, inflammatory cell infiltration, neuronal loss and disintegration, glial cell hypertrophy and hyperplasia, cyst formation and other phenomena, all of which were milder than those in the SCI only group. There was no bleeding, cysts or glial scars in the sham group (Fig. 1).

### GFAP and NF-200 Immunofluorescence

**Fig. 2:**
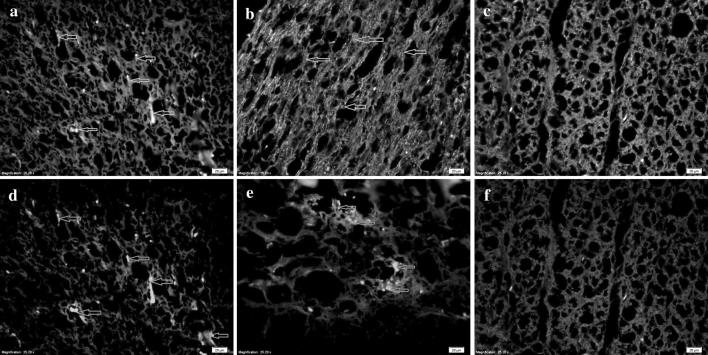
Expression of GFAP and neurofilament protein 200 (NF-200) at 28 days after spinal cord injury detected by immunofluorescence (×100). *Panels 1–3*: GFAP-positive cells (*arrows*) in the non-telomerase reverse transcriptase (SCI only), TERT siRNA, and sham groups. *Panels 4–6*: NF-200 positive cells (*arrows*) in the SCI only, TERT siRNA, and sham group

Image-ProPlus 6.0 software was used to quantify immunofluorescence images. The GFAP expression at each time point was significantly higher in the SCI only group (87 %) than in the TERT siRNA group (67 %) and sham group (2 %). At 28 days after injury, scar formation stabilized in the TERT siRNA and SCI only groups, while there was no scar formation in the sham group. NF-200 expression was gradually reduced and remained stable until day 28 in the SCI only group. There were no NF-200-labeled cells in the spinal cord glial scar and cavity at day 56 after spinal cord injury. NF-200 expression at each time was significantly lower in the SCI only group than the TERT siRNA group, while there was no change in the sham group (Fig. 2).

### TERT and GFAP Expression by Western Blotting Assay

Telomerase reverse transcriptase and GFAP expressions in the SCI only group were significantly higher than that in the TERT siRNA (*P* < 0.01; *χ* ^*2*^ = 67.722–96.521, *P* < 0.05) and sham (*χ* ^*2*^ = 159.017–167.773, *P* < 0.01; *χ* ^*2*^ = 68.526–95.461, *P* < 0.05) groups at the different time points. At 28 days after spinal cord injury, TERT and GFAP expression in the SCI only group reached a peak of 1.217 ± 0.072 and 19.40 ± 0.55, respectively. The TERT siRNA group showed a higher GFAP expression than the sham group (χ^2^ = 88.312–121.593, *P* < 0.05). There was no change in GFAP expression in the sham group (Table 1).

**Table 1:** TERT and GFAP protein expression in the three groups at different times

Time (days)	TERT				GFAP	
	SCI only (n = 5)	TERT siRNA (n = 5)	Sham (n = 5)	SCI only (n = 5)	TERT siRNA (n = 5)	Sham (n = 5)
1	0.274 ± 0.005^b^	0.081 ± 0.007^b^	0.072 ± 0.007^c^	1.98 ± 0.15^a^	1.10 ± 0.13^b^	0.46 ± 0.05^c^
3	0.386 ± 0.004^a^	0.092 ± 0.004^b^	0.073 ± 0.006^c^	6.08 ± 0.23^a^	3.40 ± 0.45^b^	0.48 ± 0.04^c^
5	0.435 ± 0.005^a^	0.028 ± 0.007^b^	0.072 ± 0.008^c^	9.04 ± 0.35^a^	7.05 ± 0.52^b^	0.46 ± 0.05^c^
7	0.557 ± 0.006^a^	0.091 ± 0.009^b^	0.074 ± 0.005^c^	12.30 ± 0.45^a^	9.62 ± 0.71^b^	0.46 ± 0.05^c^
14	0.757 ± 0.021^a^	0.042 ± 0.004^b^	0.075 ± 0.004^c^	17.50 ± 0.50^a^	14.47 ± 0.37^b^	0.46 ± 0.05^c^
28	1.217 ± 0.072^a^	0.065 ± 0.007^ab^	0.074 ± 0.005^c^	19.40 ± 0.55^a^	16.64 ± 1.02^b^	0.46 ± 0.05^c^
42	0.660 ± 0.011^a^	0.078 ± 0.008^b^	0.073 ± 0.006^c^	16.60 ± 1.14^a^	13.54 ± 0.52^b^	0.44 ± 0.05^c^
56	0.180 ± 0.004^a^	0.083 ± 0.004e^b^	0.074 ± 0.006^c^	12.60 ± 1.14^a^	8.826 ± 0.43^b^	0.48 ± 0.040^c^

Data are expressed as mean ± SD

*TERT* telomerase reverse transcriptase, *GFAP* glial fibrillary acidic protein

^a^ *P* < 0.05, comparison between TERT siRNA and sham group

^b^ *P* < 0.05, comparison between TERT siRNA and SCI only group

^c^ *P* < 0.05, comparison between SCI only and sham group

### mRNA Expression of TERT and GFAP Detected by Reverse Transcription-PCR

In the SCI only group, TERT and GFAP mRNA were expressed at 1 day after injury, peaked at 28 days, and then gradually decreased and remained stable at 56 days. The mRNA expression of TERT and GFAP was markedly higher in the SCI only group than the TERT siRNA and sham groups (*P* < 0.05). There were no obvious changes in TERT and GFAP mRNA expression in the TERT siRNA and sham groups (Table 2).

**Table 2:** TERT and GFAP mRNA expression in the three groups at different times

Time (days)	TERT mRNA			GFAP mRNA		
	SCI only (n = 5)	TERT siRNA (n = 5)	Sham (n = 5)	SCI only (n = 5)	TERT siRNA (n = 5)	Sham (n = 5)
1	3.43 ± 0.95^a^	1.91 ± 0.83^b^	1.05 ± 0.33^c^	3.20 ± 0.84^a^	2.10 ± 0.63^b^	1.03 ± 0.30^c^
3	3.90 ± 0.57^a^	1.94 ± 0.81^b^	1.07 ± 0.39^c^	4.17 ± 0.57^a^	2.40 ± 0.85^b^	1.04 ± 0.33^c^
5	5.68 ± 0.90^a^	2.11 ± 0.93^b^	1.11 ± 0.55^c^	5.95 ± 0.66^a^	4.05 ± 0.79^b^	1.05 ± 0.40^c^
7	7.46 ± 0.78^a^	2.19 ± 0.99^b^	1.11 ± 0.62^c^	7.51 ± 0.67^a^	5.26 ± 0.82^b^	1.06 ± 0.42^c^
14	9.23 ± 0.78^a^	2.40 ± 0.94^b^	1.12 ± 0.62^c^	9.30 ± 1.11^a^	6.63 ± 0.78^b^	1.07 ± 0.49^c^
28	13.34 ± 0.87^a^	2.56 ± 0.71^b^	1.16 ± 0.69^c^	13.69 ± 1.0^a^	10.69 ± 1.1^b^	1.04 ± 0.28^c^
42	9.42 ± 0.86^a^	2.30 ± 0.82^b^	1.10 ± 0.52^c^	9.51 ± 0.98^a^	6.72 ± 0.92^b^	1.03 ± 0.25^c^
56	4.48 ± 1.08^a^	2.20 ± 0.74^b^	1.04 ± 0.32^c^	6.20 ± 1.27^a^	2.36 ± 0.84^b^	1.04 ± 0.30^c^

Data are expressed as mean ± SD

*TERT* telomerase reverse transcriptase, *GFAP* glial fibrillary acidic protein

^a^ *P* < 0.05, comparison between TERT siRNA and sham group

^b^ *P* < 0.05, comparison between TERT siRNA and SCI only group

^c^ *P* < 0.05, comparison between SCI only and sham group

### Correlation between TERT and Glial Scar Formation

**Fig. 3:**
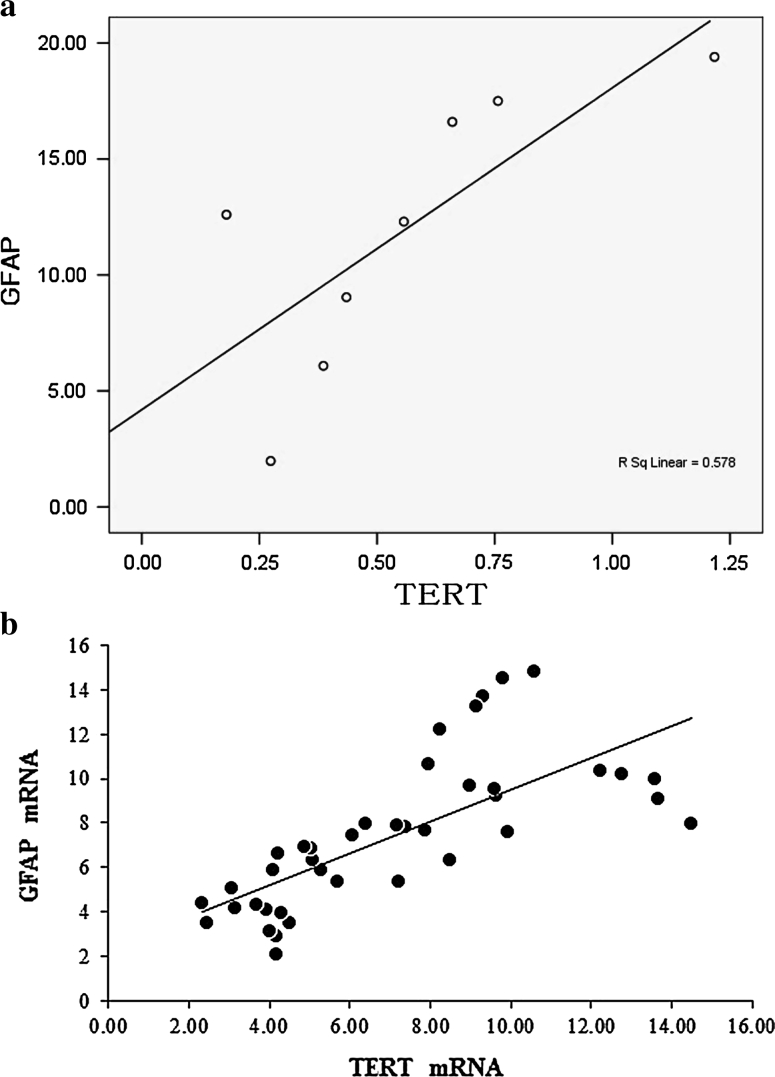
**a** Correlation between TERT and GFAP expression in the glial scar in the SCI only group. Spearman’s rank correlation test showed a positive correlation between TERT and GFAP protein expressions (r = 0.765, *P* < 0.01). **b** Correlation between TERT and GFAP mRNA expression in the glial scar in the SCI only group. Spearman’s rank correlation test showed a positive correlation between TERT and GFAP mRNA expression (r = 0.722, *P* < 0.01)

**Fig. 4:**
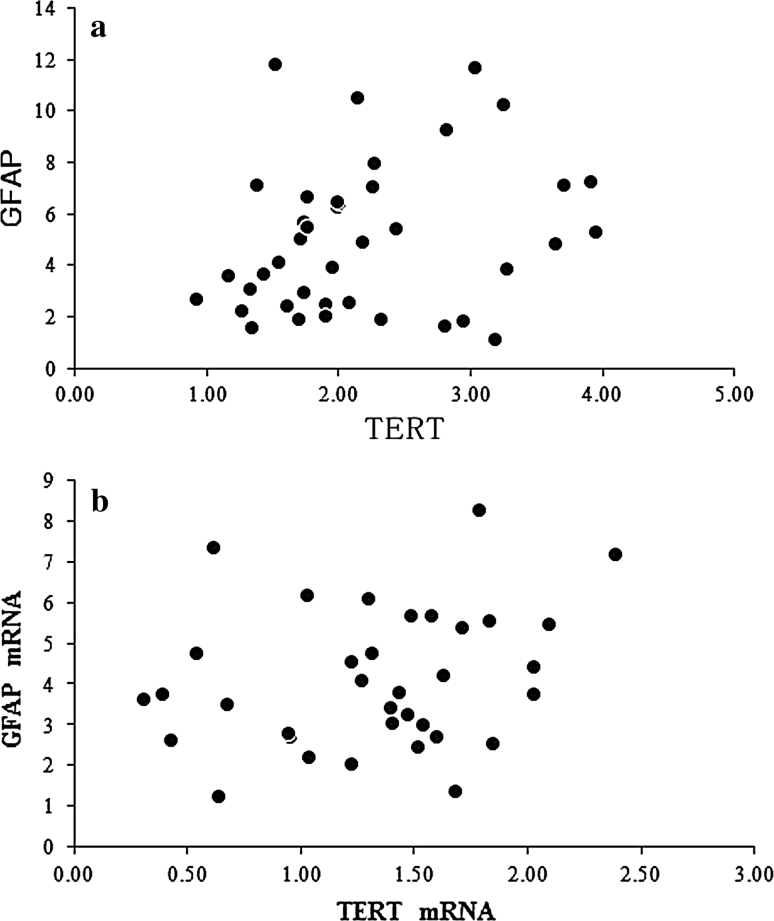
**a** Correlation between TERT and GFAP expression in the glial scar in the sham group. Spearman’s rank correlation test showed no correlation between TERT and GFAP protein expression (r = 0.208, *P* = 0.121). **b** Correlation between TERT and GFAP mRNA expression in the glial scar in the sham group. Spearman’s rank correlation test showed no correlation between TERT and GFAP mRNA expressions (*r* = 0.206, *P* = 0.180)

In the SCI only group, GFAP and TERT were positively expressed at 1 day after injury, peaked at 28 days, and then gradually decreased and remained stable at 56 days. Western blotting showed that TERT and GFAP protein expression showed a linear relationship in the SCI only group (r = 0.765, *P* < 0.01; Fig. 3a), which is consistent with the results of RT-PCR (r = 0.722, *P* < 0.01; Fig. 3b). However, there was no obvious linear relationship in the sham group (Western blotting: r = 0.208, *P* = 0.121; RT-PCR: r = 0.206, *P* = 0.180; Fig. 4).

## Discussion

After spinal cord injury, the activation of astrocytes in the injury zone and local microenvironment changes lead to the formation of glial scars, which can inhibit the growth of axons [12]. In recent years, the use of transplantation methods have markedly progressed the treatment of spinal cord injury, although restoration of spinal cord function remains to be achieved. The main reason for this is that current treatments fail to absolutely inhibit glial scar formation after spinal cord injury. In addition, the causes of glial scar formation are not fully understood.

Glial scar formation after spinal cord injury is a complex pathophysiological process that involves tissue edema, inflammation, local ischemia, glutamate receptor hyper-activation, lipid peroxidation, calcium overload, free radical damage, astrocyte activation, and apoptosis [13]. In the SCI only group, there were few hemorrhagic foci in the spinal cord gray and white matter, severe damage to the structure of the spinal cord, dissolved gray matter neurons, a large necrotic area, and cyst formation. A great amount of axons and vacuoles could be seen in the white matter, and nerve fibers were disordered. Hemorrhagic foci practically disappeared in the spinal cord gray matter and white matter by day 28 after injury, and the structure of the spinal cord was damaged to a severe extent. There was further death of gray matter neurons, which formed a large number of vacuoles. In addition, inflammatory infiltration was alleviated, and a large number of glial cells surrounded the cavity to form a dense glial scar.

Telomerase reverse transcriptase is a critical catalytic subunit of the telomerase enzyme complex and regulates telomerase activity, including cell growth and survival [14, 15]. TERT is involved in the activation of telomerase and prolongs cell life by protecting the telomere. In addition, TERT has multiple non-telomere length-dependent effects that are closely associated with cell activation, proliferation, and apoptosis [6, 9, 16], such as the regulation of intracellular Ca^2+^ distribution, mitochondrial function, energy metabolism, growth factor secretion, and apoptotic gene expression [17–21]. Herein, we focus on the biological effects of TERT through which TERT regulates glial scar formation after spinal cord injury. Previous studies have shown that glial scar formation after spinal cord injury is mainly mediated by astrocyte activation. GFAP, the most important cytokine secreted by activated astrocytes, promotes scar formation and inhibits axonal regeneration. In the present study, GFAP expression was gradually reduced in the SCI only group at day 28 after spinal cord injury and remained stable until day 56, while TERT expression was normal. Western blotting and RT-PCR showed that TERT and GFAP protein expressions had a linear relationship in the SCI only group (r = 0.765, *P* < 0.01; r = 0.722, *P* < 0.01, respectively), while there was no obvious linear relationship in the sham group (r = 0.208, *P* = 0.121; r = 0.206, *P* = 0.180, respectively). These findings suggest that TERT mRNA was involved in the activation during the formation of glial scars in the injured spinal cord. It was found that the process of astrocyte activation mainly appears as changes in Ca^2+^ concentrations [22], while TERT is important for the regulation of Ca^2+^ distribution and transmembrane transport [19, 23]. Therefore, we believe that TERT may activate astrocytes by regulating Ca^2+^ concentration, thus contributing to the formation and development of the glial scar. However, the specific pathway and mechanism require further study.

Current methods to inhibit TERT expression include antisense technology, RNA interference, and ribozyme technology [24]. On the basis of previous studies, we designed an antisense nucleotide targeting astrocyte TERT mRNA, to suppress TERT mRNA expression. Negative- and false-positive tests showed that the inhibition rate of the constructed plasmid vector was 81.05 ± 7.65 %, based on which PBCA-NPs-TERT was confirmed to effectively inhibit TERT expression.

In summary, we demonstrated that TERT exhibited dynamic expression in the spinal cord glial scar, similar to GFAP, which is used to measure glial scar severity, but there were no changes in TERT and GFAP expressions in the sham group. Unfortunately, inhibition of TERT mRNA expression failed to significantly reduce the number of glial scars, indicating that TERT is not the only contributor to glial scar formation. Further studies are required to examine other molecules contributing to the formation of the glial scar in the spinal cord, thereby deepening our understanding of the etiology of spinal cord injury and promoting the treatment of spinal cord injury.
